# Perioperative and oncologic outcomes of transperitoneal versus retroperitoneal laparoscopic radical nephrectomy for large-volume renal carcinoma (> 7 cm): a systematic review and pooled analysis of comparative outcomes

**DOI:** 10.1186/s12957-023-02967-1

**Published:** 2023-03-09

**Authors:** Li Wang, Kun-peng Li, Ying Liu, Shan Yin, Ping-yu Zhu

**Affiliations:** 1 Department of Urology, Affiliated Hospital of North Sichuan Medical College, Nanchong, 637000 China; 2 Department of Urology, Affiliated Hospital of Lanzhou University Second Hospital, Lanzhou, 730030 China

**Keywords:** Laparoscopic nephrectomy, Kidney neoplasms, Transperitoneal, Retroperitoneal, Meta-analysis

## Abstract

**Background:**

Recently, there has been a significant amount of debate concerning the question of whether laparoscopic surgery should be performed transperitoneally or retroperitoneally for treating large renal tumors.

**Aim:**

The purpose of this research is to conduct a comprehensive review and meta-analysis of the previous research on the safety and efficacy of transperitoneal laparoscopic radical nephrectomy (TLRN) and retroperitoneal laparoscopic radical nephrectomy (RLRN) in the treatment of large-volume renal malignancies.

**Methods:**

An extensive search of the scientific literature was carried out utilizing PubMed, Scopus, Embase, SinoMed, and Google Scholar in order to locate randomized controlled trials (RCTs) and prospective and retrospective studies that compared the effectiveness of RLRN versus TLRN in the treatment of for large renal malignancies. For the purpose of comparing the oncologic and perioperative outcomes of the two techniques, data were taken from the research studies that were included and pooled together.

**Results:**

A total of 14 studies (five RCTs and nine retrospective studies) were incorporated into this meta-analysis. The overall RLRN had an association with significantly shorter operating time (OT) (MD [mean difference]: − 26.57; 95% CI [confidence interval]: − 33.39 to − 19.75; *p* < 0.00001); less estimated blood loss (EBL) (MD: − 20.55; CI: − 32.86 to − 8.23; *p* = 0.001); faster postoperative intestinal exhaust (MD: − 0.65; CI: − 0.95 to − 0.36; *p* < 0.00001). The terms of length of stay (LOS) (*p* = 0.26), blood transfusion (*p* = 0.26), conversion rate (*p* = 0.26), intraoperative complications (*p* = 0.5), postoperative complications (*p* = 0.18), local recurrence rate (*p* = 0.56), positive surgical margin (PSM) (*p* = 0.45), and distant recurrence rate (*p* = 0.7) did not show any differences.

**Conclusions:**

RLRN provides surgical and oncologic results similar to TLRN, with potential advantages regarding shorter OT, EBL, and postoperative intestinal exhaust. Due to the high heterogeneity among the studies, long-term randomized clinical trials are required to obtain more definitive results.

**Supplementary Information:**

The online version contains supplementary material available at 10.1186/s12957-023-02967-1.

## Introduction

Renal cell carcinoma (RCC) is responsible for almost 3% of all cancers, and approximately one-third of these cases are large and locally advanced [1]. Although minimally invasive techniques have become a widely accepted approach for the management of small renal masses, their application for the treatment of large renal tumors remains controversial. Since laparoscopic and robotic approaches techniques are less invasive and have been shown to significantly enhance patient outcomes, they are increasingly being used in the management of large renal masses. However, these approaches may be limited by the size and location of the tumor as well as by the degree to which the tumor is close to adjacent structures [2, 3].

As such, the use of an intraperitoneal or retroperitoneal approach may be necessary for the safe performance of the nephrectomy of these larger tumors. The former approach involves dissecting the mass from the anterior aspect of the kidney and may result in longer operative time and increased risk of bowel injury and adhesions, while the latter approach involves dissecting the mass from the posterior aspect of the kidney and may result in shorter operative time. The transperitoneal approach may be beneficial for patients with complex anatomy or severe comorbidities [4].

A complete systematic review and meta-analysis of the relevant research were carried out in order to provide a more in-depth and accurate comparison of the relative efficacy of these two different laparoscopic techniques. Our findings may provide physicians with the opportunity to make decisions based on data regarding the most effective method of treating large renal masses.

## Methods

This study adhered to the standards specified in PRISMA (Preferred Reporting Items for Systematic Reviews and Meta-Analysis) [ 5] and was prospectively registered in the PROSPERO database (CRD42022383039).

### Search strategy

The research for the literature included in the systematic review was performed independently by two reviewers (WL and LY). Relevant electronic databases searched include PubMed, Scopus, Embase, SinoMed, and Google Scholar. The data obtained from the literature were before December 1, 2022. The combined search phrases relevant to patients and interventions used in creating the search string were as follows: [(renal OR kidney) AND (carcinoma OR cancer OR malignancy) AND (laparoscope OR laparoscopic OR endoscopes) AND (transperitoneal OR retroperitoneal)]. In addition, we had no language requirement for inclusion and cross-checked the reference list to prevent the omission of relevant information. Any disagreements were resolved through amicable negotiation and when consensus could not be reached, a third reviewer (YS) was consulted in order to make a final decision.

### Study selection

The PICOS principles were used for the inclusion of relevant literature. P (patient): All patients diagnosed with unilateral renal tumors > 7 cm in size; I (intervention): undergoing RLRN; C (comparator): TLRN was performed as a comparator; O (outcome): perioperative variables and complications are assessed; S (study type): randomized controlled studies (RCTs) and prospective or retrospective case–control studies. The exclusion criteria were as follows: (1) single-arm studies that did not compare RLRN and TLRN; (2) conference abstracts, reviews, case reports, and unpublished studies; (3) no complete or available data from the study.

### Data extraction and quality assessment

Using a predetermined Excel spreadsheet, the two reviewers (KP and YS) independently extracted the data that follows: (1) demographics and clinical features: study design, patient number, gender, age, body mass index (BMI), preoperative creatinine, American Society of Anesthesiologists (ASA) score, tumor laterality, and size, clinical stage, and tumor pathology; (2) surgical outcomes: operative time (OT), estimated blood loss (EBL), length of hospital stay (LOS), blood transfusion; conversion rate, postoperative intestinal exhaust, and intra- and postoperative complications; (3) oncology-related outcomes: local recurrence rate, positive surgical margin (PSM), and distant recurrence rate.

The quality assessment of both randomized controlled was performed by the modified Jadad scale with scores ranging from 0 to 7, with studies considered high-quality if they got a score of 4 or higher and case–control studies by Newcastle–Ottawa Scale (NOS) with scores ranging from 0 to 9, and the research methodology design was considered acceptable if it had a score of 6 or higher. Additionally, each study’s evidence was assessed using Oxford Evidence-based Medicine Center criteria [6]. Two reviewers assessed the quality and evidence of the study and resolved the differences through discussion.

### Statistical analysis

This meta-analysis was conducted utilizing Review Manager Version 5.0 (The Cochrane Collaboration, Oxford, London, UK) and STATA 14.0 (StataCorp, College Station, TX). Dichotomous and continuous variables were expressed using odds ratios (ORs) and weighted mean differences (WMDs), respectively. Data from some studies reporting only medians, quartiles, or extreme ranges were converted to means and standard deviations (SDs) utilizing the data conversion tables provided by Luo et al. [7] and McGrath et al. [8]. All outcomes were reported with 95% CIs. Statistical heterogeneity between studies was tested by the chi-square test and inconsistency (*I* ^2^). The combined effect size results are expressed by the *Z* test and are statistically significant at *p* < 0.05. Random effects are applied when *I* ^2^ > 50%. If not, a fixed effects model is employed [9]. Sensitivity analysis is performed to assess the robustness between studies through an exclusion-by-exclusion method. In addition, confounding factors such as year of publication, region, and study design between studies were assessed by meta-regression tests.

### Publication bias

When the number of studies was greater than ten, we used a funnel plot combined with an Egger’s regression test to synthetically evaluate possible publication bias [10]. The presence of publication bias was unconsidered when *P* > 0.05.

## Result

### Baseline characteristics

**Fig. 1:**
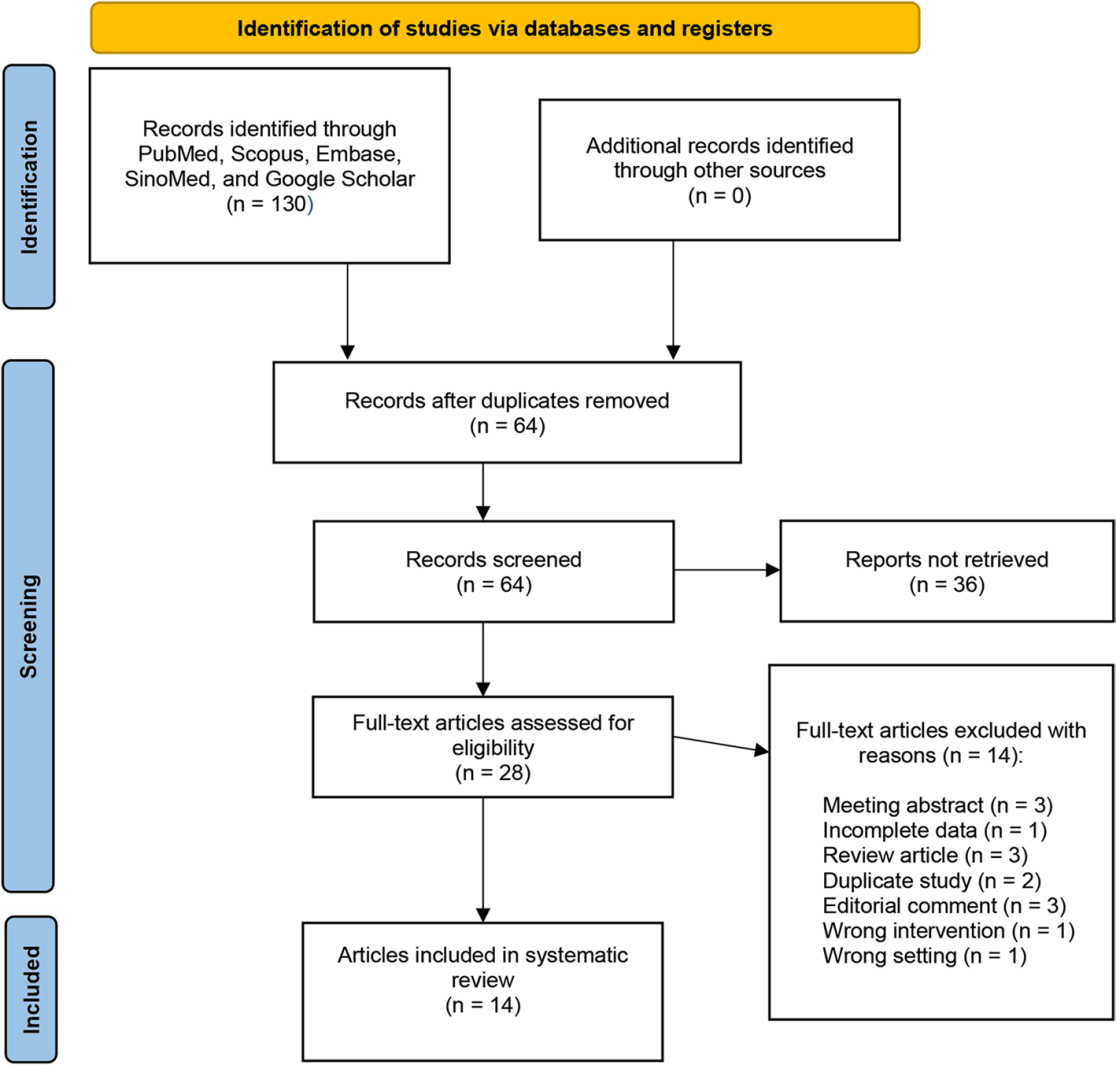
PRISMA flowchart

Based on the initial screening of the search strategy, we identified 130 relevant publications, and after eliminating duplicates and reviewing titles, abstracts, and full texts, 14 controlled studies were included. Figure 1 shows the PRISMA flow chart**.** The studies spanned from 2004 to 2021, with five randomized controlled studies [11–15] and nine retrospective case–control studies [16–24], with twelve [11–15, 17–22, 24] from China, one [23] from Korea, and one [16] from the USA in the study population. Within the 1093 patients in this meta-analysis, 573 (52.4%) and 520 (47.6%) were treated with RLRN and TLRN, respectively. Table 1 summarizes the relevant features and variables of the study. No statistically significant difference was found between groups regarding male (*p* = 0.83), BMI (*p* = 0.58), age (*p* = 0.99), tumor laterality (*p* = 0.63), tumor size (*p* = 0.07), ASA score (*p* = 0.14), and baseline creatinine (*p* = 0.19) (Table 2).

**Table 1:** Overview of collected studies

Reference	Study design	Approach	Patients(n)	Comparability	Clinical stage	Tumor pathology	Quality score	Level of evidence	Follow-up (month)
Yin (2021) [24]	Retrospective	RLRN	58	1, 2, 3, 4, 5	All is T2 and T3	Clear cell: 47. Papillary: 7. Chromophobe: 3. Collecting duct: 1	6 ^b^	4	23.3 ± 4.4
		TLRN	58			Clear cell: 51. Papillary: 10. Chromophobe:7			
Chu (2019) [15]	RCT	RLRN	36	1, 2, 3, 4, 5, 7	All is T2 and T3	NA	5 ^a^	2b	NA
		TLRN	36						
Huang (2018) [13]	RCT	RLRN	40	1, 2, 3, 4, 5, 7	NA	NA	5 ^a^	2b	NA
		TLRN	40						
Chen (2018) [14]	RCT	RLRN	32	1, 2	NA	NA	4 ^a^	2b	NA
		TLRN	32						
Zhang (2017) [22]	Retrospective	RLRN	53	1, 2, 3, 4, 5	All is T2 and T3	NA	5 ^b^	4	NA
		TLRN	52						
Kim (2017) [23]	Retrospective	TLRN	30	1, 2, 3, 4, 5	T2:18 T3:6 T4:6	Clear cell: 26. Other: 4	6 ^b^	4	NA
		RLRN	34		T2:17 T3:10 T4:7	Clear cell: 31. Other:3			
Wu (2016) [21]	Retrospective	TLRN	30	1, 2, 3, 4, 5, 6, 7	All is T2 and T3	NA	5 ^b^	4	NA
		RLRN	30						
Song (2016) [20]	Retrospective	RLRN	43	1, 2	All is T2 and T3	NA	5 ^b^	4	NA
		TLRN	43						
Chen (2016) [19]	Retrospective	TLRN	35	1, 2, 3, 4, 5, 6, 7	T2:15 T3:20	NA	5 ^b^	4	6
		RLRN	38		T2:32 T3:6				
Qin (2015) [12]	RCT	TLRN	50	1, 2, 4, 5	NA	NA	4 ^a^	2b	NA
		RLRN	50						
Huang (2015) [11]	RCT	TLRN	40	1, 2, 4, 5	T2:15 T3:25	NA	5 ^a^	2b	NA
		RLRN	40		T2:26 T3:14				
Xu (2014) [18]	Retrospective	RLRN	42	1, 2, 3, 4, 5	T2:35 T3:7	Clear cell:42	5 ^b^	4	NA
		TLRN	26		T2:21 T3:5	Clear cell: 26			
Yang (2013) [17]	Retrospective	TLRN	23	1, 2, 3, 4, 5, 6, 7	T2:10 T3:13	Clear cell: 23	6 ^b^	4	NA
		RLRN	37		T2:28 T3:9	Clear cell: 31. Papillary: 2. Chromophobe: 2. Collecting duct: 1. Eosinophilic: 1			
Steinberg (2004) [16]	Retrospective	RLRN	40	2, 3, 5	All is T2, T3 and T4	NA	5 ^b^	4	NA
		TLRN	25						

*RCT* randomized controlled trial, *RLRN* retroperitoneal laparoscopic radical nephrectomy, *TLRN* transperitoneal laparoscopic radical nephrectomy, *NA* not available

^a^Modified Jaded scale scores

^b^Newcastle–Ottawa scale scores. Comparability: 1 = gender (M/F); 2 = age (years); 3 = body mass index; 4 = tumor laterality (L/R); 5 = tumor size (cm); 6 = American Society of Anesthesiologists score; 7 = baseline creatinine (umol/L)

**Table 2:** The demographics of the studies

Variable	No. of studies with available data	WMD/OR	95% CI	*p* value
Male (*n*)	13	1.03	(0.8, 1.32)	0.83
BMI (kg/m^2^)	9	− 0.09	(− 0.41, 0.23)	0.58
Age (years)	13	0.01	(− 0.69, 0.7)	0.99
Tumor laterality (L/R)	11	0.94	(0.72, 1.22)	0.63
Tumor size (cm)	11	− 0.15	(− 0.31, 0.01)	0.07
ASA score	3	0.09	(− 0.03, 0.22)	0.14
Baseline creatinine (umol/L)	5	0.72	(− 0.35, 1.79)	0.19

*WMD* weighted mean difference, *OR* odds ratio, *Cl* confidence interval, *BMI* body mass index, *ASA* American Society of Anesthesiologists score

### Assessment of quality

Randomized controlled trials were all of high quality, only three retrospective controlled studies were of high quality, and overall, study quality was generally moderate or below.

### Surgical outcomes

#### Meta-analysis of perioperative effectiveness

**Fig. 2:**
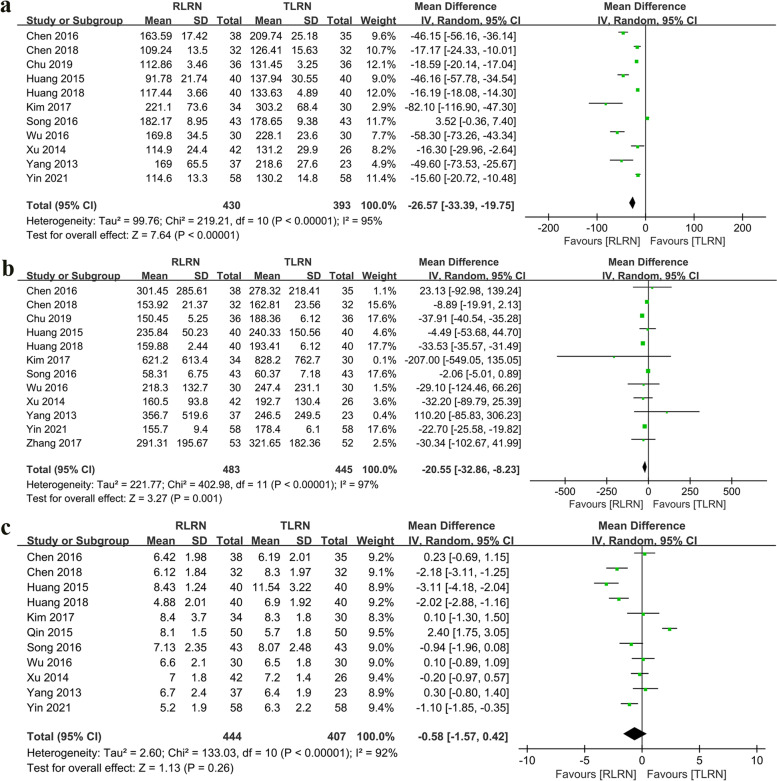
Forest plot of perioperative outcomes: **a** Operative time (min). **b** Estimated blood loss (ml). **c** Length of stay (day)

Eleven studies [11, 13–15, 17–21, 23, 24] reported operating time (OT) in a total of 823 patients (430 RLRN vs. 393 TLRN), and the pooled results showed RLRN significantly reduced OT (WMD: − 26.67 min; 95% CI: − 33.39, − 19.75; *p* < 0.0001). Twelve studies [11, 14, 15, 17–24] evaluated estimated blood loss (EBL), including 928 patients (483 RLRN vs. 445 TLRN), and the pooled results showed that RLRN has lower EBL compared to TLRN (WMD: − 20.55 min; 95% CI: − 32.86, − 8.23; *p* = 0.001). Eleven studies [11–14, 17–21, 23, 24] analyzed hospital stay, 851 in total (444 RLRN vs. 407 TLRN), and the meta-analysis did not show significant differences in LOS between RLRN and TLRN (OR: − 0.58; 95% CI: − 1.57, 0.42; *p* = 0.26) (Fig. 2).

**Fig. 3:**
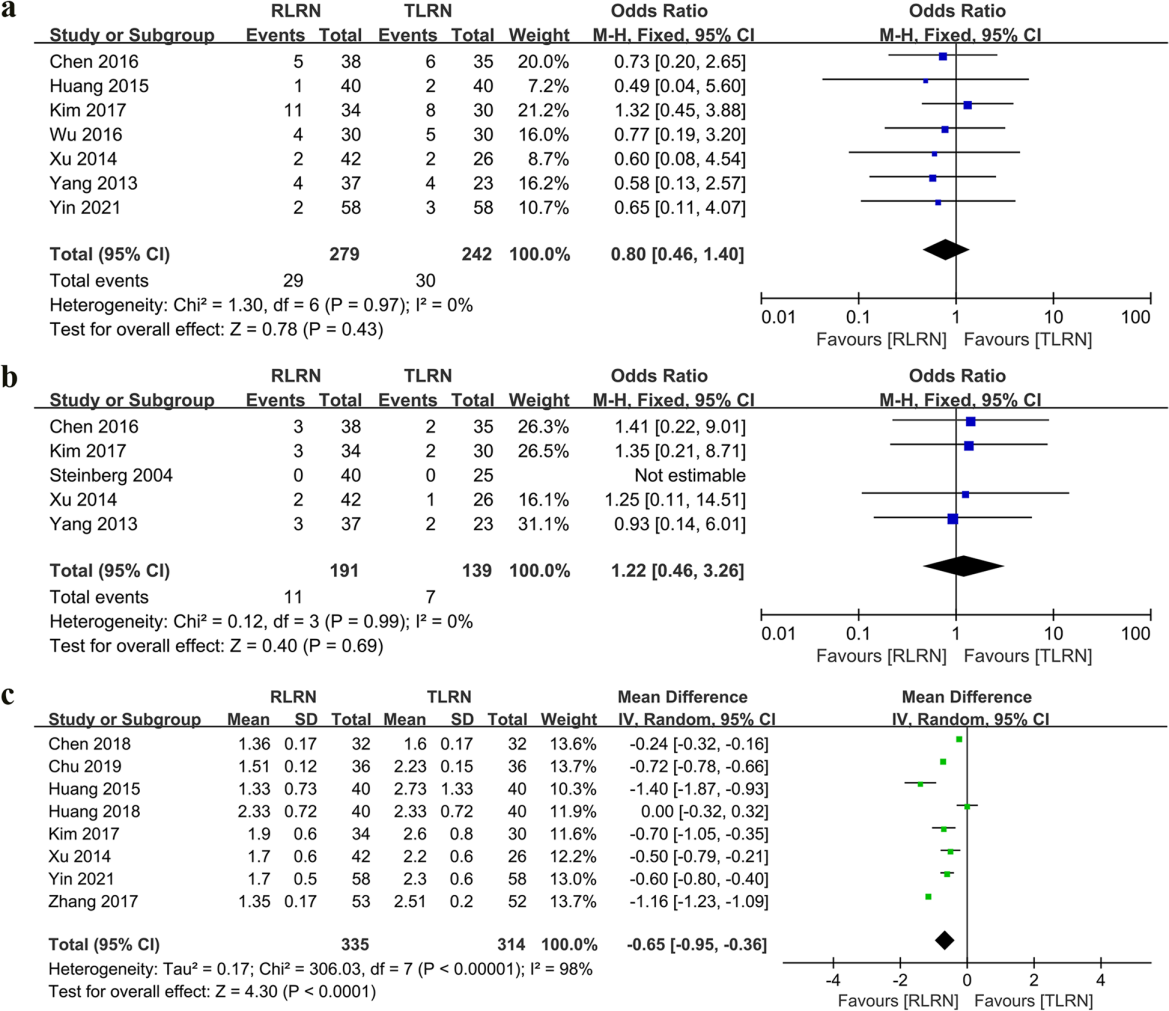
Forest plot of perioperative outcomes: **a** Blood transfusion. **b** Conversion rate. **c** Postoperative intestinal exhaust (day)

Seven studies [11, 17–19, 21, 23, 24] reported transfusion rates. A comparison of the two groups (297 RLRN vs. 242 TLRN) indicates that RLRN and TLRN have similar transfusion rates (OR: 0.8; 95% CI: 0.46, 1.4; *p* = 0.43). Five studies [16–19, 23] reported conversion rates, and RLRN patients converted 5.7% (11/191), while TLRN patients converted 5% (7/139). The meta-analysis showed that RLRN and TLRN had comparable conversion rates (OR: 1.22; 95% CI: 0.46, 3.26; *p* = 0.69). The pooled analysis of eight studies [11, 13–15, 18, 22–24] showed a shorter postoperative period of intestinal exhaust after surgery in patients with RLRN (WMD: − 0.65 day; 95% CI: − 0.95, − 0.36; *p* < 0.0001) (Fig. 3).

#### Meta-analysis of complications

**Fig. 4:**
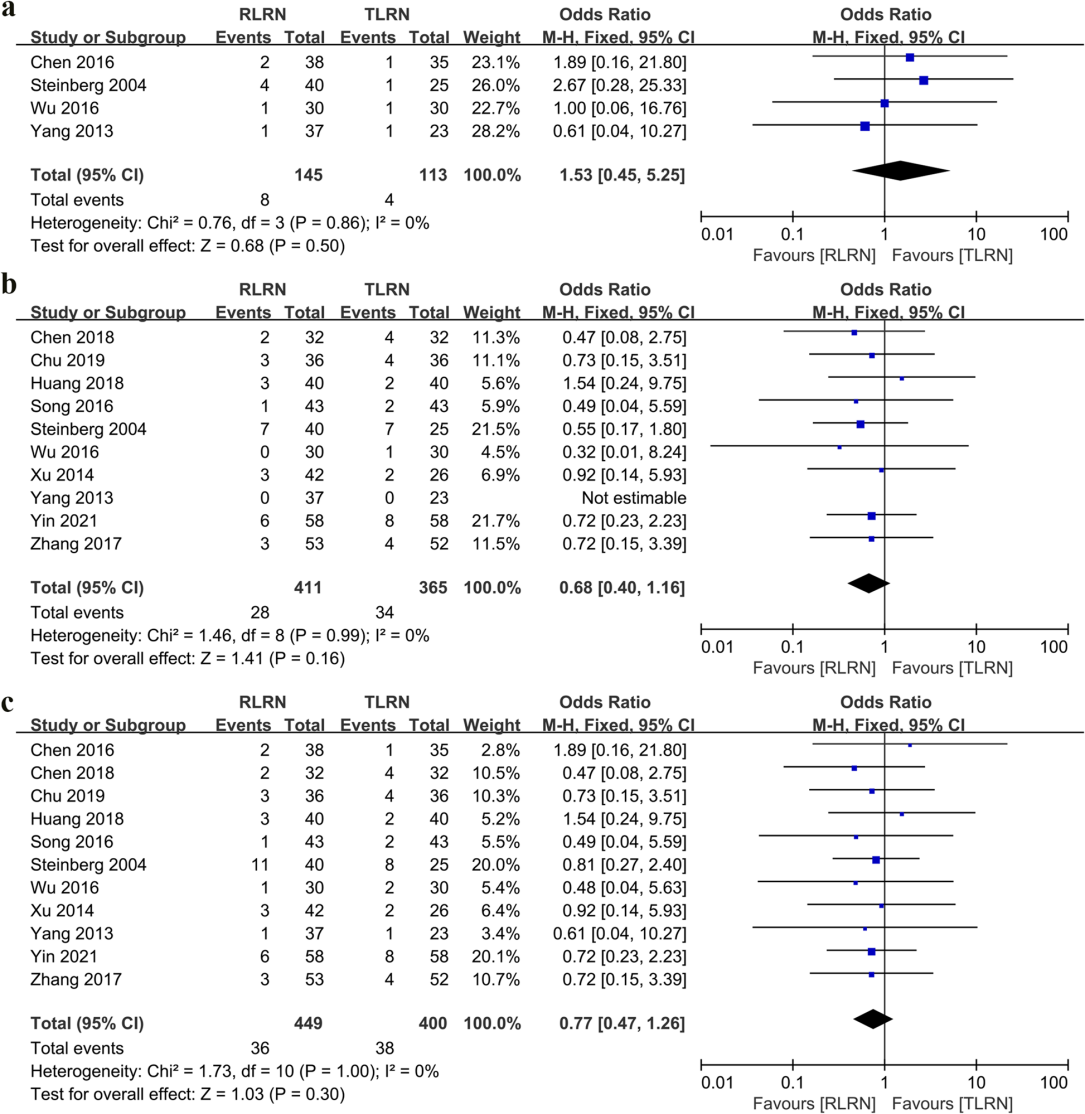
Forest plot of complications: **a** Intraoperative complications. **b** Postoperative complications. **c** Overall complication

In terms of intraoperative complications, there was no statistically significant difference between the two different surgical approaches (OR: 1.53; 95% CI: 0.45, 5.25; *p* = 0.5). Similarly, the postoperative complications had similar outcomes (OR: 0.68; 95% CI: 0.4, 1.16; *p* = 0.16). The overall complication rates for RLRN were 8.01% (36 out of 449 cases) and 9.5% (38 out of 400 cases) for the TLRN group, respectively (Fig. 4).

#### Meta-analysis of oncologic outcomes

**Fig. 5:**
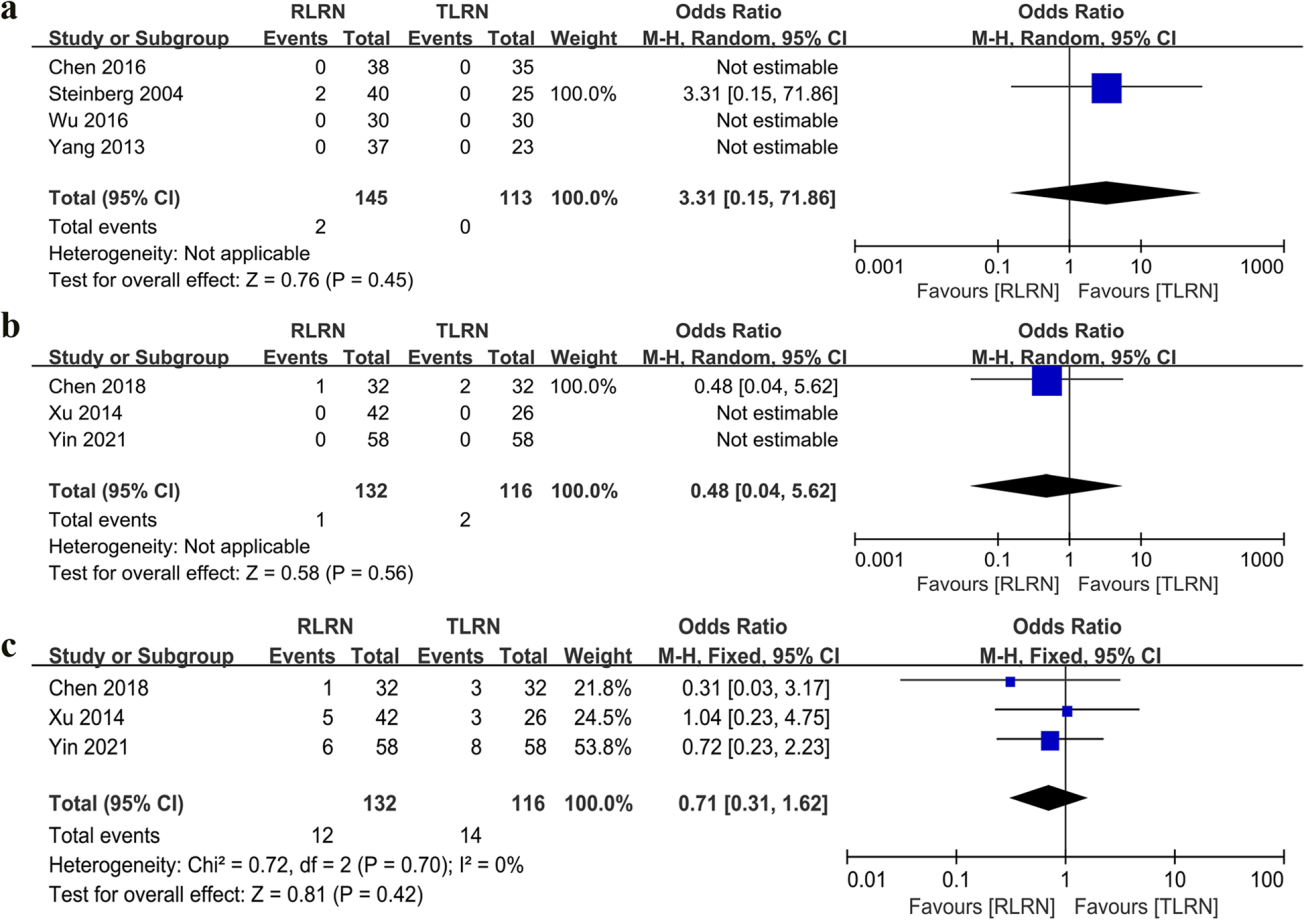
Forest plot of oncological outcomes: **a** Positive surgical margin. **b** Local recurrence rate. **c** Distant recurrence rate

Four studies [16, 17, 19, 21] reported PSM, and no statistically significant differences were found between RLRN and TLRN (*p* = 0.45). Similarly, the cumulative analysis of the three studies [14, 18, 24] did not show statistically significant differences between RLRN and TLRN in terms of local recurrence (*p* = 0.56) and distant metastasis (*p* = 0.7) (Fig. 5).

### Heterogeneity

Some outcome indicators (OT, EBL, LOS, and postoperative intestinal exhaust) were highly heterogeneous, we included literature of largely moderate or low quality, and we attempted to eliminate some confounding factors, such as region, year of publication, and type of study design by meta-regression analysis. No obvious source of heterogeneity was found (*p* > 0.05) (Additional file 1). Of course, we cannot ignore the bias introduced by small sample studies [25].

### Sensitivity analysis and publication bias

Due to the high heterogeneity of some results (OT, EBL, LOS, and postoperative intestinal exhaust), a sensitivity analysis was applied to the target parameters in order to obtain robust and convincing conclusions. The effect size was recalculated by leave-one-out methods, which shows that the results are robust (Additional file 2). Using funnel plot evaluations, no evidence of publication bias was identified (OT, EBL, LOS), and Egger’s regression test (all *P* > 0.05) (Additional file 3).

## Discussion

Our findings suggest that RLRN may be a promising option for the treatment of large-volume renal tumors, and further research is warranted to investigate its efficacy and safety.

### Surgical outcomes

Our study results indicate that RLRN has a shorter OT than TLRN. This is likely due to the retroperitoneal approach providing easier access to the tumor site and rapid exposure of the renal hilum and vasculature. Conversely, the intra-abdominal approach, although providing a larger working space, entails the challenge of manipulating and separating intra-abdominal structures to expose the tumor, thereby leading to a longer OT [26].

The cumulative analysis of several studies indicates that the retroperitoneal approach is associated with an approximate 20.55-mL reduction in estimated blood loss (EBL) compared to the intra-abdominal approach. This reduction can be attributed to the early exposure of the renal hilum, which permits the ligation of renal vessels and thus minimizes the risk of bleeding from surrounding organs that is associated with the intra-abdominal approach when large renal tumors invade the nearby vasculature [27].

Although the statistics show that RLRN is more advantageous in terms of intraoperative blood loss, we must be cautious about this result given the interference of preoperative differences in patient hemoglobin levels, surgeon experience, and choice and error of measuring instruments.

The LOS for a patient is primarily determined by the knowledge and beliefs of the specialist doctor and the capacity of the hospital. The concept of rapid postoperative recovery can reduce LOS for minimally invasive surgery [28]. Our meta-analysis shows that LOS is significantly unaffected by the surgical approach. Piramide et al. [29] summarized several studies concerning robotic-assisted partial nephrectomy (RAPN) and observed that operative time, surgeon experience, and postoperative protocol adherence were influential in predicting whether patients could be discharged overnight after the procedure. However, additional research is required for the confirmation of this finding and a better understanding of the factors influencing LOS for patients undergoing minimally invasive surgery.

Through the mastery of laparoscopic surgical skills and ligation of parasitic vessels in large renal tumors, our meta-analysis shows no significant difference in intraoperative blood transfusion and conversion to open surgery between the two laparoscopic surgical approaches. Most previous studies showed that laparoscopic access allows traction and mobilization of the bowel, particularly in large renal tumors, providing a wider view and operative space. Our cumulative analysis showed that patients who underwent TLRN had longer postoperative bowel recovery and exhaust time compared to those who underwent RLRN, approximately 0.65 days. However, Nambirajan et al. [30] found that patients undergoing laparoscopic surgery had a higher proportion of tolerating oral intake on the first postoperative day (100% vs. 75%), although this result should be interpreted with caution due to the use of robotic assistance and most of tumors in stage T1 of the study population.

### Complications

Our pooled analysis of existing studies suggested the presence of no statistically significant difference in terms of perioperative and postoperative complications between (RLRN) and (TLRN). Despite slightly higher perioperative complications of RLRN (5.5% vs. 3.5%), postoperative complications were lower (6.8% vs. 9.3%). The most common complications are vascular damage, urinary tract injury, and wound infection. Vascular damage may lead to hematuria and decreased renal function. Urinary tract injury may present as urinary fistula and hydronephrosis. Wound infection usually occurs near the trocar sites. In addition, some other complications have been reported, such as renal artery thrombosis, intra-abdominal bleeding, and pneumothorax [31, 32]. These findings indicate that RLRN and TLRN are both effective in terms of producing good clinical outcomes and preventing complications in the long term. There is a necessity for additional research to confirm these findings and ascertain the most appropriate surgical approach for different clinical scenarios.

### Survival

Due to the limitations of the sample size and the lack of long-term follow-up data, our cumulative analysis only reports on the rates of positive surgical margins, local recurrence, and distant metastases. The results did not indicate statistically significant differences between the abdominal and retroperitoneal approaches. The Yin [ 24] study compared overall survival (18.3 months vs. 19.1 months) and progression-free survival (16 months vs. 16.8 months) of RLRN and TLRN using log-rank tests and again found no statistically significant differences.

Our results suggest that RLRN may be a superior option in treating large renal masses compared to TLRN. One potential explanation for the superior outcomes of the laparoscopic retroperitoneal approach is the improved visualization of the renal hilum and surrounding structures. The retroperitoneal approach may allow for the complete removal of perirenal fat, which may reduce the risk of local recurrence. However, the retroperitoneal approach requires an additional incision on the flank, which can increase the risk of postoperative hernia. Moreover, the retroperitoneal approach may be technically challenging for surgeons with limited experience in laparoscopic surgery. Furthermore, the retroperitoneal approach may be correlated to a longer learning curve compared to the transperitoneal approach. In conclusion, there is still some debate as to which route of treatment is more effective for large renal neoplasms. While the transperitoneal approach provides complete tumor excision, the retroperitoneal approach is less invasive and has fewer major complications. Ultimately, the choice of route should be left to the surgeon, taking into account the patient’s specific clinical situation.

### Limitations

Our research includes a variety of limitations, all of which need to be taken into consideration before drawing any conclusions from the findings. To begin, we only included a restricted number of research studies, which may have limited the ability to produce reliable statistical results. Furthermore, the quality of included studies was variable. Most studies were observational in nature, which is subject to bias compared to randomized controlled trials. Regression analysis failed to detect high heterogeneity in some outcomes and therefore caution is needed when analyzing study results. Thirdly, the studies included in our analysis had a relatively short duration of follow-up, which may be inadequate to evaluate long-term outcomes such as cancer-specific survival and overall survival. Finally, the laparoscopic transperitoneal and retroperitoneal approaches may have been conducted by different surgeons with diverse levels of experience and expertise. This may have affected the accuracy of our comparison between the two approaches.

## Conclusions

Our analysis suggests that RLRN and TLRN have similar surgical and oncological outcomes. Furthermore, RLRN may have potential advantages, including shorter OT, lower EBL, and faster postoperative bowel function recovery. Despite the high heterogeneity among the studies, additional evidence is required to verify the robustness of the results, preferably from long-term follow-up prospective randomized clinical trials.

## Supplementary Information

**Additional file 1.** Meta-regression analysis of surgical outcomes.

**Additional file 2.** Sensitivity analysis of surgical outcomes.

**Additional file 3.** Forest plot to explore publication bias.

## Data Availability

The article and the supplementary material contain all of the datasets that were produced by this investigation.

## Abbreviations

RLRN: Retroperitoneal laparoscopic radical nephrectomy
TLRN: Transperitoneal laparoscopic radical nephrectomy
RCTs: Randomized controlled trials
OT: Operating time
MD: Mean difference
WMDs: Weighted mean differences
ORs: Odds ratios
CI: Confidence interval
EBL: Estimated blood loss
LOS: Length of stay
PSM: Positive surgical margin
RCC: Renal cell carcinoma
PRISMA: Preferred Reporting Items for Systematic Reviews and Meta-Analysis
BMI: Body mass index
ASA: American Society of Anesthesiologists
NOS: Newcastle-Ottawa Scale
RAPN: Robotic-assisted partial nephrectomy
