# Supplementary figures and images for: Perioperative and oncologic outcomes of transperitoneal versus retroperitoneal laparoscopic radical nephrectomy for large-volume renal carcinoma (> 7 cm): a systematic review and pooled analysis of comparative outcomes
Source: World J Surg Oncol. 2023 Mar 9;21:86. doi: 10.1186/s12957-023-02967-1 (PMC9997016; doi:10.1186/s12957-023-02967-1)

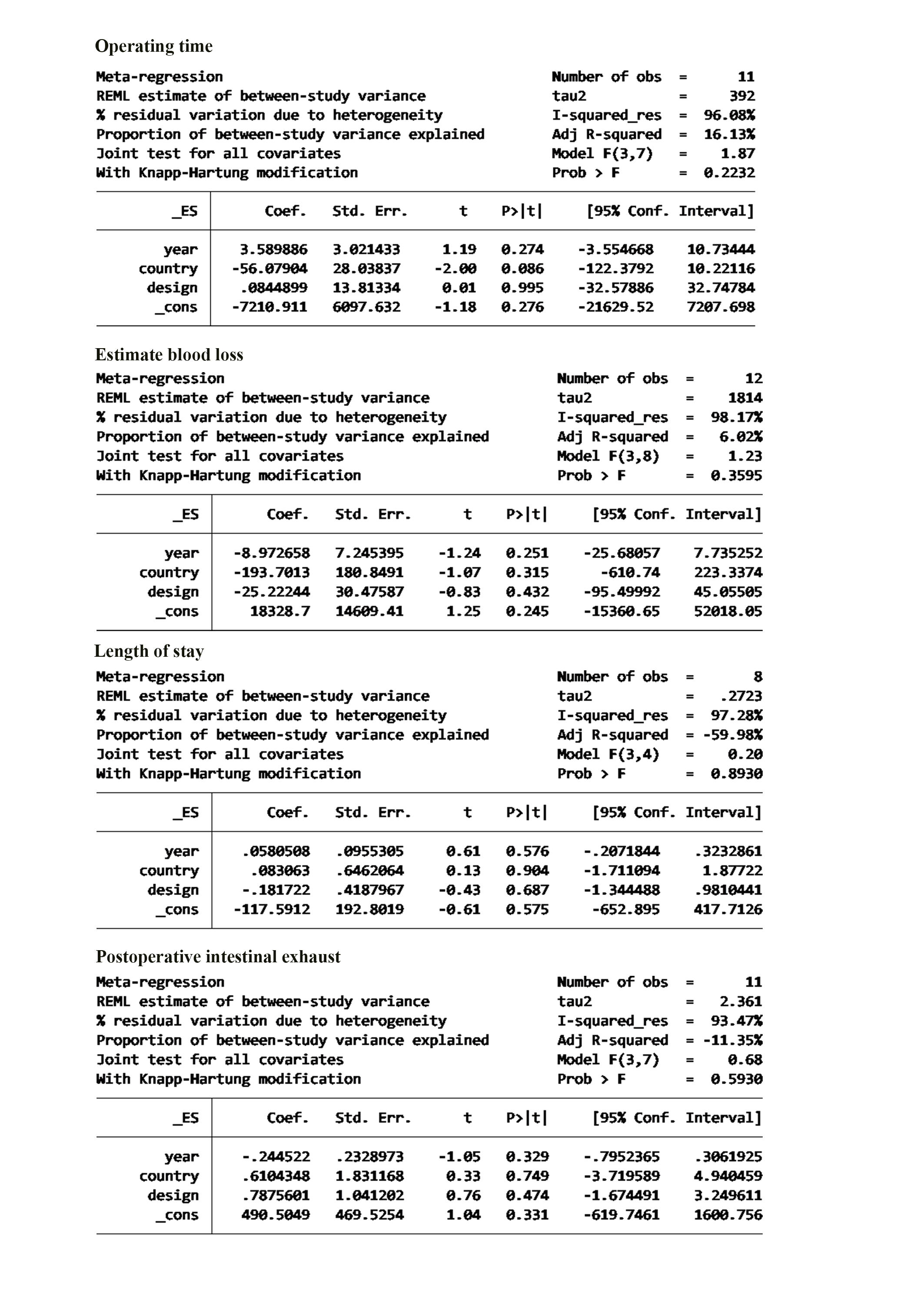

Supplement: Supplementary file 1 — Additional file 1. Meta-regression analysis of surgical outcomes. [file 12957_2023_2967_MOESM1_ESM.tif]

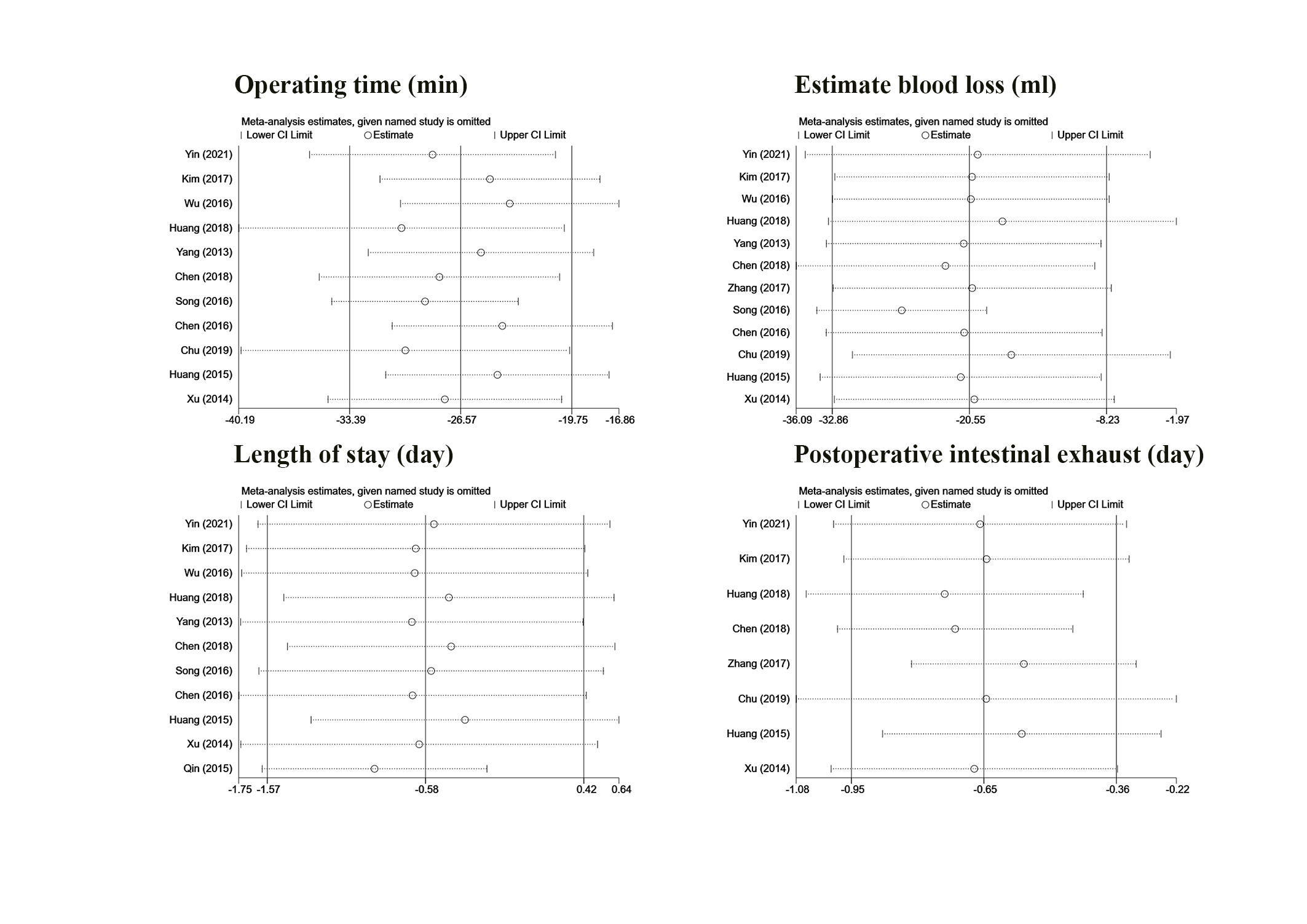

Supplement: Supplementary file 2 — Additional file 2. Sensitivity analysis of surgical outcomes. [file 12957_2023_2967_MOESM2_ESM.tif]

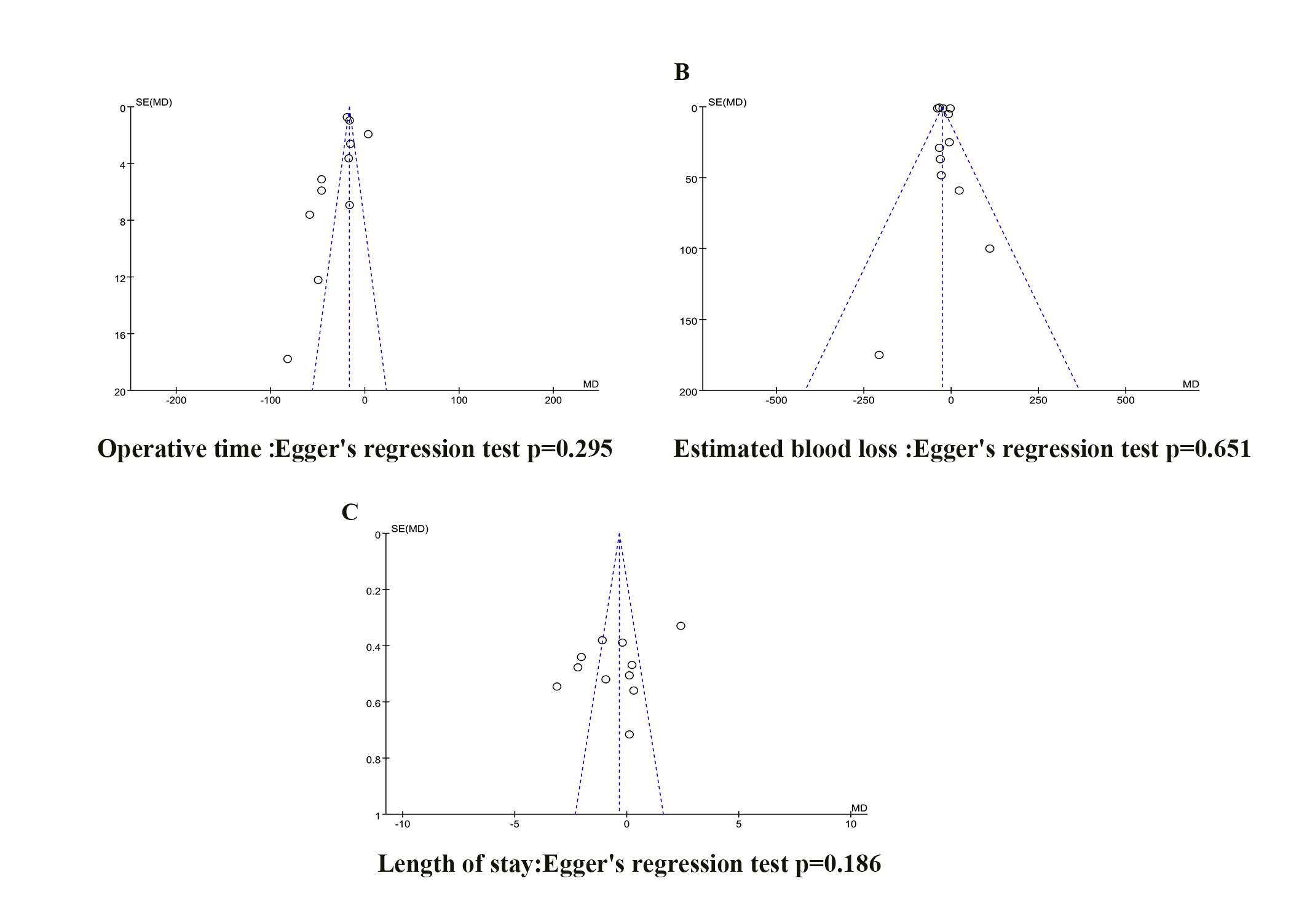

Supplement: Supplementary file 3 — Additional file 3. Forest plot to explore publication bias. [file 12957_2023_2967_MOESM3_ESM.tif]
